# The context matrix: Navigating biological complexity for advanced biodesign

**DOI:** 10.3389/fbioe.2022.954707

**Published:** 2022-08-23

**Authors:** Camillo Moschner, Charlie Wedd, Somenath Bakshi

**Affiliations:** Control Group, Department of Engineering, University of Cambridge, Cambridge, United Kingdom

**Keywords:** synthetic biology, context, biodesign, function-centric, design of experiments, standardisation

## Abstract

Synthetic biology offers many solutions in healthcare, production, sensing and agriculture. However, the ability to rationally engineer synthetic biosystems with predictable and robust functionality remains a challenge. A major reason is the complex interplay between the synthetic genetic construct, its host, and the environment. Each of these contexts contains a number of input factors which together can create unpredictable behaviours in the engineered biosystem. It has become apparent that for the accurate assessment of these contextual effects a more holistic approach to design and characterisation is required. In this perspective article, we present the context matrix, a conceptual framework to categorise and explore these contexts and their net effect on the designed synthetic biosystem. We propose the use and community-development of the context matrix as an aid for experimental design that simplifies navigation through the complex design space in synthetic biology.

## 1 Introduction

Synthetic biology is defined by the design and construction of biological systems for useful purposes. This approach typically follows a *Design-Build-Test-Learn* cycle. Various tools and pipelines have been created to standardise, enhance, or automate this construction cycle or specific segments of it (Carbonell et al., 2018; Jessop-Fabre and Sonnenschein, 2019). However, the design process in particular remains elusive to standardisation approaches. This is attributed to various factors, in particular the difficulty of choosing between vast numbers of design principles that could yield the desired function. Therefore, design typically relies on the experimenter’s knowledge of the biosystem they are working with.

Acquisition of this knowledge typically relies on mining the literature. However, this is a time-consuming, labour-intensive and inefficient process. From a materials perspective, this bottleneck led to a focus on the creation and characterisation of parts and repositories like the *iGEM Registry of Standardised Biological Parts* or the *SynBioHub* (Canton et al., 2008; Kwok, 2010; McLaughlin et al., 2018). However, it has become clear that the performance of these parts is highly context-dependent. For example, the same construct-host combination will likely exhibit different behaviours in different growth media, pH or temperatures (Boo et al., 2019; Chory et al., 2021). Similarly, in a fixed environment, the same construct can display dramatically different performance in different hosts. Many context-defining factors have been identified and characterised (Cardinale and Arkin, 2012) but have so far lacked a connection into one user-friendly framework for design and troubleshooting.

**FIGURE 1:**
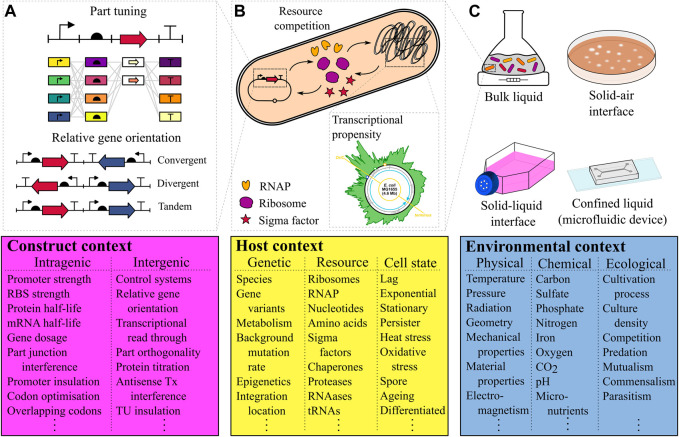
The three contexts of an engineered biosystem’s function **(A)** Construct context includes factors intrinsic to the design of the synthetic genetic circuit which can affect performance, and can be broadly divided into intra and inter-transcription unit construct contexts. An example of an intra-TU context is the composition of the construct itself, which can be tuned with different parts to achieve different outputs. Relative gene orientation is an example of an inter-TU context, which can significantly affect expression (Yeung et al., 2017). **(B)** The host context concerns all factors where the host organism affects the performance of the biosystem, and can be divided into the contexts of genetic factors, resource competition and the state in which the cell is growing. Resource competition (top, middle panel), is the phenomenon of a synthetic circuit competing with the host genome for shared resources. For genome-integrated circuits differing transcriptional propensities (indicated by the height of the green region) specify how the location of the circuit in the chromosome will affect performance (Scholz et al., 2019). **(C)** The environmental context in which the biosystem operates is defined by physical, chemical and ecological factors (reducing to just physical and chemical factors for cell-free systems). A selection of cultivation processes are illustrated, the differences between which are likely to have significant effects on gene expression in individual cells and shape the growth of the population as a whole. Acroynms: TU = transcription unit. RNAP = RNA polymerase. RBS = Ribosomal binding site.

Here we present the “context matrix,” a database of previously encountered input factors categorised based on the contexts of either synthetic genetic construct, host or environment (Figure 1) which is intended to help experimenters identify their key input factors and enable the full utilisation of biological complexity. We first explain the structure of the matrix, then use specific examples to highlight each context’s potential implication in system performance, how contexts overlapping with each other create emergent behaviours, and finally discuss how this information could be utilised to inform advanced design strategies.

## 2 The context matrix

**FIGURE 2:**
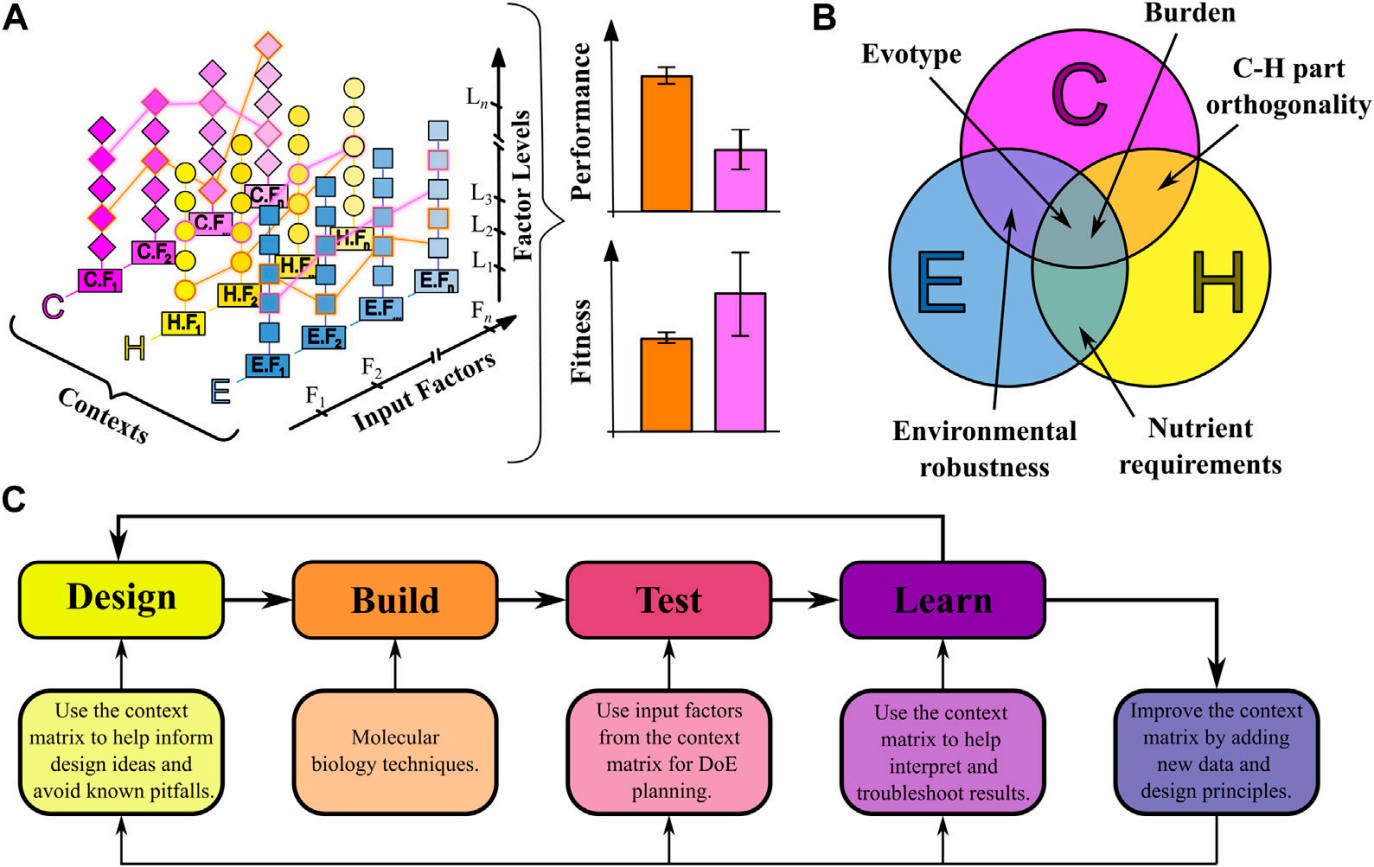
The context matrix and its applications. **(A)** A representation of the context matrix. The three primary contexts (construct = C, host = H, environment = E), are each composed of a number of input factors (C.F_1_, E.F_2_, … ), which are described in Figure 1. The input factors are further subdivided into levels (C.F_1_. L_1_, C.F_1_. L_2_, … ). Continuous input factors (such as temperature or glucose concentration) can take any feasible finite level, whereas categorical input factors (such as species or gene orientation) are restricted to discrete values. The chosen combination of all input factors (orange or pink outlines) completely defines the context of an engineered biosystem, and can be thought of as an input landscape. Each input landscape will produce an input-output mapping to outputs such as performance and fitness. **(B)** Emergent properties arising from overlapping contexts. **(C)** Integrating the context matrix into the *Design-Build-Test-Learn* cycle for context-aware synthetic biology. DoE = Design of Experiments.

The context matrix is a multi-dimensional list of input factors that enables decision making about what factors are important for the function of the engineered biosystem because of their combined effect on the output performance (Figure 2A), and helps to quickly identify factors previously unknown to the experimenter. Our approach considers a function-centric rather than the more traditional construct-centric view of synthetic biology. The biosystem function is seen in the contexts of synthetic construct, host, and environment composition. These contexts have been chosen based on their relatively independent preparation in the design process. For example, a genetic construct can be created outside of a host, and a host can be grown in various different environmental conditions. Each context is further divided into unique factors which can be quantitative or qualitative in nature. Understanding the current position of the engineered biosystem within this context space is essential for the design of novel biosystems, and for understanding and troubleshooting their failure modes. The input space from the context matrix maps to a specific, emergent phenotypic output state, such as performance of the engineered biosystem, or the fitness of the host (Figure 2A).

### 2.1 Synthetic construct context

The first component of the context matrix, the synthetic genetic construct, captures contextual effects and failure modes intrinsic to the design of the synthetic system. This is closely associated with the term “genetic circuit design” in synthetic biology (Brophy and Voigt, 2014). The factors of the construct context can conceptually be divided into intra- and intergenic categories.

In the first instance, part tuning of a transcription unit (TU) might be considered which often includes the testing of various promoter and ribosome binding site strength levels (Figure 1A). This represents an example of intragenic design. Transcription read-through from one TU into another based on terminator choice is an intergenic design factor that can be utilised to create “transcriptional valves” (Tarnowski and Gorochowski, 2022). While this strategy purposefully utilises intergenic interactions, a lot of attention has focused on the creation and characterisation of orthogonal parts to avoid intergenic cross-talk. For example, Meyer et al. (2019) showed the extensive part engineering required to create 12 transcription factors that can be used in the same cell without interfering with one another. Brophy and Voigt (2014) provides a holistic review of principles of genetic circuit design. Recently, various other potential construct design input factors have been identified. For example, the relative orientation of two mutually-repressive TUs has been shown to yield up to a 400% difference in maximal expression between convergent and divergent TU arrangements (Figure 1A) and has been associated with DNA supercoiling events inside the construct itself (Yeung et al., 2017). Furthermore, advances in sequencing technology have enabled the identification of cryptic, anti-sense promoters inside coding sequences of a multi-TU logic gate that interfere with the desired biosystem function (Gorochowski et al., 2017). While this exemplifies anti-sense transcription interference as a failure mode, for a different engineered biosystem it can be harnessed as a construct input factor to tune gene expression (Brophy and Voigt, 2016). If such tuning is not desired, codon optimisation strategies or double terminators between TUs can be used as input factors to mitigate anti-sense transcription interference.

### 2.2 Host context

The second component of the context matrix concerns the host, and captures advantages and disadvantages behind different host choices and their implications for the performance of an engineered biosystem. Input factors in this context include host-specific structural, metabolic or pre-existing genetic characteristics and includes knowledge of global gene regulatory mechanisms (Figure 1B). One of the first host input factors to consider is what domain of life and specific species is most suitable for a given application. For example, eukaryotic cell engineering is often preferred for the production of humanized therapeutic proteins due to their already existing post-translational modification machinery (Dumont et al., 2016). More recently, bioengineers have started to harness subcellular compartmentalisation as a means to eliminate metabolic pathway cross-talk and utilise organelle-specific micro-environments for particular biochemical reactions (Hammer and Avalos, 2017; Gassler et al., 2020; Grewal et al., 2021).

Host input factors are closely associated with the notion of “host-aware synthetic biology” (Boo et al., 2019). For example, synthetic biologists have increasingly become aware of the effects of plasmid-based versus genome-integrated constructs on output performance and have extensively investigated genome location effects (Block et al., 2012; Bryant et al., 2014; Sauer et al., 2016; Englaender et al., 2017; Goormans et al., 2020). Analysis of genome wide RNA polymerase activity lead to the mapping of “transcriptional propensities”, a measure of a genomic region’s likelihood to be transcribed (Figure 1B), across the entire 4.6 Mb *E. coli* genome (Scholz et al., 2019). These transcriptional propensities are the product of a complex interplay between nucleoid-associated proteins and the chromosome leading to actively and passively silenced regions, similar to the well-known mechanisms of chromosome compaction in eukaryotic heterochromatin (Wang et al., 2011). Knowledge of transcriptional propensities have been utilised to identify genome locations well-suited to accommodate heterologous gene circuits (Goormans et al., 2020; Park et al., 2020).

Another crucial host input factor is the cell state in which it is supposed to be used. Most synthetic biology studies using *E. coli*, for example, test synthetic constructs in nutrient-rich, exponential growth phase. The resulting cell state is known to heavily utilise the growth-related *σ* ^70^ global transcriptional regulator and hence the majority of characterised promoters are based on this sigma factor. More recently other cell states have been utilised in the design of engineered biosystems that operate in stationary (Gefen et al., 2014; Jaishankar and Srivastava, 2020), stressed (Rodrigues and Rodrigues, 2018) and even spore states (Mohsin et al., 2021; Quijano and Sahin, 2021). Consideration of the cell state will be vital in the ongoing transition towards real-world applications of engineered biosystems.

### 2.3 Environmental context

The third component of the context matrix concerns the environment, capturing physical and chemical variables, and ecological interactions (Figure 1C). To create a particular functional output from an engineered biosystem, the environmental composition must either be carefully selected and maintained, or the biosystem should be designed such that its function is robust to changes in environmental context.

As designers, we have the opportunity to precisely control the initial chemical composition of a biosystem. Complex media such as lysogeny broth (LB) (Bertani, 1951, 2004) and trypticase soy broth (TSB) (FDA, 2001) are frequently used in bacterial studies. However, these complex nutrient broths include poorly defined components (for example, LB contains yeast extract and protein digests), and therefore the use of complex media makes it impossible for researchers to know the precise chemical composition of their biosystem environment. This calls into question the reproducibility of physiological studies utilising LB or other complex media, and significant differences in gene expression between different brands of LB have been observed (Sridhar and Steele-Mortimer, 2016).

This problem has long been recognised in microbiology. Neidhardt et al. (1974) published a fully defined media formulation optimised for the growth of enterobacteria, noting that the ill-defined components and autoclave sterilisation (as opposed to filter sterilisation) used in complex media preparation can introduce variability and uncertainty in results. Wherever possible, fully defined media should be used in characterisation studies, such that the chemical context of an engineered biosystem can be controlled and optimised. A recent systematic mapping of the concentration of phosphorus, carbon and nitrogen sources in a defined media to the growth rate and protein expression of *E. coli* cultures revealed media formulations which give high protein expression without sacrificing growth (Chory et al., 2021). This exemplifies the importance of the environmental context in achieving the desired biosystem function. This reasoning extends beyond bacteria, and media optimisation through measurements of the changing quantities of metabolites present in the culture has been used to double antibody titres from mammalian cells (Sellick et al., 2011).

In many applications precise control of the environmental composition may not be attainable, and a biosystem function which is robust to environmental perturbations is desirable. For example, various applications do not allow control of temperature, a key physical input factor which can affect biosystem function due to its effect on the rates of biochemical reactions (Peterson et al., 2007). A synthetic gene circuit known as the dual-feedback oscillator exhibits a period which decreases with increasing temperatures (Stricker et al., 2008). For this system, a temperature-stable period was achieved by using a temperature-sensitive *lac* repressor protein (Hussain et al., 2014), demonstrating how a simple change in the construct context can give a robust functional performance in different environmental contexts. Another example of a synthetic construct yielding environmentally robust performance is the repressilator, a different genetic oscillator (Elowitz and Leibier, 2000). Potvin-Trottier et al. (2016) found that removing degradation tags and using protein titration in an improved design created an oscillator with a stable period of 14 cell generations across a range of temperatures. This design was later verified to be robust to changes in growth-phases using a microfluidic setup for complex growth conditions (Bakshi et al., 2021) and was also applied to quantify bacterial growth dynamics in the mouse gut, demonstrating the robustness of the desired function in an unknown and changing environmental context (Riglar et al., 2019).

## 3 Emergent properties of context effects

While the context matrix is generally divided into the three contexts of synthetic construct, host and environment, some properties of an engineered biosystem cannot be usefully fit into just one of these contexts but rather emerge from the interplay between two or even all three contexts simultaneously (Figure 2B).

One well-studied example is the emergence of genetic burden. In synthetic biology, burden is generally defined as an unnatural load on the host by the synthetic construct (Ceroni et al., 2015; Borkowski et al., 2016; Lee et al., 2016; Gorochowski et al., 2019). Many studies have identified resource competition between construct and host as the cause of burden (Kim et al., 2020). Qian et al. (2017) showed that a simple two node repression cascade can be rescued through a decrease in plasmid-copy number and RBS strengths of the circuit, indicating burden effects due to ribosome limitation (Figure 1B). Furthermore, the effects of the system’s environment have been shown to be equally important: By modifying rather than deleting endogenous genes Keren et al. (2016) showed that burden effects for the same expression level of a gene significantly varied in different media. While genetic burden is not a design input factor itself but an emergent property of the engineered biosystem, awareness of the phenomenon has been cleverly utilised to create burden-driven feedback loops limiting the burden effects themselves (Ceroni et al., 2018).

Interference between construct and host factors has also been recognised as an important emergent property of engineered biosystems (Del Vecchio et al., 2018). For example, Cookson et al. (2011) showed that two synthetic proteins which are both targeted for degradation by the *E. coli* ClpXP machinery can saturate the degradation system. This results in protein queuing of the synthetic proteins and stochastic accumulation of one of the proteins, resulting in failure of the designed function of the biosystem. Potvin-Trottier et al. (2016) identified a similar specific interference effect in the repressilator. The reporter protein in this oscillator construct contained an endogenous ClpXP degradation tag that caused fluctuations in the degradation of the circuit’s repressor proteins and had to be removed to create regular oscillations. One potential mitigation strategy to avoid this specific interference is to engineer the construct to be as orthogonal as possible to the host. Various tools for orthogonal protein degradation in bacteria (Cameron and Collins, 2014) and eukaryotes (Pedone et al., 2019) have been created for this purpose.

A further important consideration for successful design is evolution. Largely due to the effects of burden, the function performed by a synthetic construct is rarely stable in a population, and will be lost over time as fitter, non-functional mutants arise (Sleight et al., 2010). Castle et al. (2021) developed a powerful conceptual framework for integrating evolutionary considerations into the design process. They define the “design type” as the engineered biosystem at the point of design. They then describe in detail the “evotype”, the set of evolutionary dispositions of the design type, and different ways in which the evotype can be shaped to change the probabilities of certain evolutionary outcomes. Consideration of the evotype is essential where prolonged or changing function is required. Efforts to properly characterise and understand contextual effects in synthetic biology will help to more completely define the design type, and should aid efforts to characterise and understand the long-term evolution of a biosystem.

## 4 Context-aware biodesign

The context matrix represents an easily navigable database of knowledge of design strategies available to an experimenter, and lists strategies that might not have been known or considered before. Its emphasis on a function-centric view means it aims to achieve the desired biosystem function while being agnostic towards any particular construct, host or environment context.

A consequence from this is that multiple engineered biosystems with the same function but very different contexts can be created. We call such systems analogous engineered biosystems. It is our goal to allow for simplified comparison between analogous systems to quickly identify the most appropriate design strategy based, for example, on available material, time constraints or even patent-protected genetic parts. This idea fits into the statistical field of Design of Experiments (DoE), in which multiple input factors are designed and tested simultaneously to identify the ones significant to the desired application (Gilman et al., 2021).

A common desired biosystem function is a simple induction system in which a gene of interest (GOI) is expressed after the cell receives a chemical or optical signal (Ong and Tabor, 2018; Groseclose et al., 2020). Traditionally this is implemented through a constitutively expressed, protein-based repressor inhibiting the GOI until the signal leads to a conformational change in the repressor, relieving its inhibition. However, when designing such a simple system the first question is which repressors to choose from. An array of part repositories and characterisation experiments allows informed decision making about what repressor characteristics are available (Meyer et al., 2019). However, thus far no database of further design strategies exists. Once a repressor has been chosen, the question emerges about how to arrange the two transcription units of GOI and repressor with respect to each other. Most induction systems desire a low leakiness in the inhibited state. Looking at the context matrix (see GitHub repository) we learn that a convergent orientation is likely to increase inhibitory effects on the GOI through supercoiling of its DNA, and therefore decreases leakiness (Yeung et al., 2017). However, if this is still not sufficient one might consider changing the construct to an RNA-based riboregulator or even a mixed RNA-plus protein-based multi-level controller which has shown reduced leakiness and improved dynamic range (Greco et al., 2021). If the desired function can be hosted in a eukaryotic organism additional design strategies for induction system improvements can include physical separation of the activating element from the GOI through protein localisation out of the nucleus (Di Ventura and Kuhlman, 2016) or designed, epigenetic-based silencing of the GOI (Sgro and Blancafort, 2020).

The limited examples presented here exemplify how even a simple induction system can benefit from various advanced design strategies and how the context matrix can help the experimenter identify which ones are suitable for their application.

## 5 Community development

We envisage the context matrix as a community-built resource to aid in the design, characterisation, understanding and standardisation of engineered biosystems and their applications.

To make continuous progress in populating the context matrix and for the field to develop a broader and deeper understanding of the role of contextual effects in synthetic biology, it is important that all relevant contextual factors are reported in studies. This should include all physical and chemical factors, some accounting of the history of the system (for example, the conditions under which cells were grown overnight prior to the experiment), and the DNA sequence data of all synthetic constructs and host genomes within the biosystem studied, except in cases where publishing such data could pose a biosecurity risk (Smith and Sandbrink, 2022). The reporting of all this data should be sufficient to allow the biosystem to be fully and accurately recreated. Even in cases where some of this information seems irrelevant, it is still worth reporting, as it may be relevant to researchers in allied fields. As the field of synthetic biology grows, complete and rigorous data reporting will help ensure results can be reproduced and understood, and will aid in standardisation efforts (Endy, 2005; Arkin, 2008, 2013). The value of standardisation in synthetic biology is demonstrated by the success of SBOL as a tool to aid the visualisation and design of synthetic biological parts (Galdzicki et al., 2014). Other standards, such as for data acquisition (Sainz De Murieta et al., 2016), have also been developed. However, there is currently no standardised way to report or characterise contextual effects in synthetic biology, and the context matrix is a step towards this goal.

The context matrix is hosted on GitHub (https://github.com/camos95/context_matrix). We welcome content suggestions (adding new input factors or expanding and clarifying existing ones) and feedback or code submissions to improve the user experience via GitHub or email.

## 6 Conclusion

Since the inception of synthetic biology, a myriad of different design strategies have been developed, utilising all biomolecules, various model and non-model organisms, and complex environmental and growth conditions. Here we present a holistic design framework for the categorisation of the learned design principles called the context matrix. As the field continues to advance, we envision the context matrix to evolve from a list of design strategies to a complete database which maps any given combination of input factors to experimental outputs. Such a database could then be queried with a design brief and return candidate designs for testing, and explanations of the mechanisms behind these recommendations. Ultimately, the context matrix aims to help facilitate synthetic biology’s transition to a more context-aware future.

## Data Availability

A continuously updated, community-driven context matrix and additional information about how to contribute to the development can be found on our GitHub page: https://github.com/camos95/context_matrix.
